# The effects of synthesis gas feedstocks and oxygen perturbation on hydrogen production by *Parageobacillus thermoglucosidasius*

**DOI:** 10.1186/s12934-024-02391-4

**Published:** 2024-05-02

**Authors:** Michael Mol, Magda Stephania Ardila, Bronwyn Ashleigh Mol, Habibu Aliyu, Anke Neumann, Pieter de Maayer

**Affiliations:** 1 School of Molecular and Cell Biology, Faculty of Science, University of the Witwatersrand, Johannesburg, 2000 South Africa; 2 Section II: Electrobiotechnology, Institute of Process Engineering in Life Science, Karlsruhe Institute of Technology, 76131 Karlsruhe, Germany

**Keywords:** *Parageobacillus thermoglucosidasius*, Water–gas shift, Hydrogen, Oxygen

## Abstract

**Background:**

The facultatively anaerobic thermophile *Parageobacillus thermoglucosidasius* is able to produce hydrogen gas (H_2_) through the water–gas shift (WGS) reaction. To date this process has been evaluated under controlled conditions, with gas feedstocks comprising carbon monoxide and variable proportions of air, nitrogen and hydrogen. Ultimately, an economically viable hydrogenogenic system would make use of industrial waste/synthesis gases that contain high levels of carbon monoxide, but which may also contain contaminants such as H_2_, oxygen (O_2_) and other impurities, which may be toxic to *P. thermoglucosidasius*.

**Results:**

We evaluated the effects of synthesis gas (syngas) mimetic feedstocks on WGS reaction-driven H_2_ gas production by *P. thermoglucosidasius* DSM 6285 in small-scale fermentations. Improved H_2_ gas production yields and faster onset towards hydrogen production were observed when anaerobic synthetic syngas feedstocks were used, at the expense of biomass accumulation. Furthermore, as the WGS reaction is an anoxygenic process, we evaluated the influence of O_2_ perturbation on *P. thermoglucosidasius* hydrogenogenesis. O_2_ supplementation improved biomass accumulation, but reduced hydrogen yields in accordance with the level of oxygen supplied. However, H_2_ gas production was observed at low O_2_ levels. Supplementation also induced rapid acetate consumption, likely to sustain growth.

**Conclusion:**

The utilisation of anaerobic syngas mimetic gas feedstocks to produce H_2_ and the relative flexibility of the *P. thermoglucosidasius* WGS reaction system following O_2_ perturbation further supports its applicability towards more robust and continuous hydrogenogenic operation.

**Supplementary Information:**

The online version contains supplementary material available at 10.1186/s12934-024-02391-4.

## Background

Increasing energy demands and dwindling fossil fuel reserves are driving the development and implementation of environmentally friendly energy production technologies, using renewable resources [1]. Hydrogen (H_2_) gas is regarded as an ideal energy carrier, with carbon neutral combustion characteristics and high energy density [2]. While H_2_ gas is currently produced almost exclusively through the reformation of non-renewable fossil fuels, various alternative renewable H_2_ gas production processes have and are currently being developed [3].

Numerous microbial taxa are capable of producing H_2_ gas from a wide spectrum of organic and inorganic feedstocks in an environmentally friendly and energetically favourable manner [4, 5]. These are subdivided into light dependent and independent processes with those incorporating dark-, photo-, and hybrid-fermentative processes having dominated mainstream research [4]. Despite yielding promising results, these processes are hindered by limitations such as cost, feedstock availability/suitability, turnover rate and scalability, that restrict their industrial application [3]. Less well-explored hydrogenogenic processes such as the biological water–gas shift (WGS) reaction, are promising alternatives to the aforementioned systems.

The reversible WGS reaction involves the oxidation of carbon monoxide to carbon dioxide and the reduction of water to molecular hydrogen at an equimolar ratio (CO + H_2_O → CO_2_ + H_2_ ∆G^O′^ =  − 20 kJ/mol CO) [6]. The potential to conduct the WGS reaction has been identified in more than twenty-five bacterial species spanning the phyla Proteobacteria, Firmicutes and Dictyoglomi and among four archaeal species in the phyla Crenarchaeota and Euryarchaeota [7]. The biological WGS reaction has been best characterised in the mesophilic proteobacterium *Rhodospirillum rubrum*, the thermophilic Firmicutes *Carboxydothermus hydrogenoformans* and *Parageobacillus thermoglucosidasius* and the hyperthermophile *Thermococcus onnurineus* in the phylum Euryarchaeota [8–11]. The reaction proceeds via a carbon monoxide dehydrogenase (CODH)/H_2_ evolving hydrogenase enzyme complex [12–14]. Both the CODH and H_2_ evolving hydrogenase enzymes are highly sensitive to O_2_, greatly restricting their industrial applicability [15, 16]. Although several hydrogenases demonstrate greater O_2_-tolerance, they exhibit poorer H_2_ gas turnover efficiencies [16]. As such, the majority of bacterial taxa capable of conducting the WGS reaction are strict anaerobes. One exception, however, is the facultatively anaerobic thermophile *P. thermoglucosidasius*, which carries a Ni-CODH/NiFe group 4a hydrogenase complex and is capable of the WGS reaction following oxygen depletion [10, 17–19]. By optimising growth medium composition and pH, fermentation temperatures and CO concentration, H_2_ gas production yields have been improved [20], but further developments towards industrial application are necessary. Key foci include progression of the process towards more continuous operation and evaluation of system efficiency when utilising gas feedstocks more reflective of industrially/commercially available waste gases. Industrial waste gasses or synthesis gases (syngas) are highly variable, typically comprising CO, H_2_ and CO_2_. Impurities including tars, particulate carbon, light hydrocarbons, inorganic compounds (sulfur-, nitrogen-, halogen- and metal-incorporated compounds) and O_2_ are also typically found at low levels [21, 22].

Here we investigated the effects of gas feedstocks, with compositions more reflective of industrial waste gases, on H_2_ gas production by *P. thermoglucosidasius* through bioassays in conjunction with GC and HPLC analysis of select gases and metabolites. Furthermore, as optimal *P. thermoglucosidasius* growth is observed under aerobic atmospheres, balancing aerobic conditions conducive to sustained biomass accumulation and anaerobic conditions obligatory for H_2_ production is necessary for more advanced continuous/semi-continuous hydrogenogenic operation. Therefore, the impacts of temporal and volumetric O_2_ perturbations (as would occur with industrial and syngas) on *P. thermoglucosidasius* DSM 6285 hydrogenogenesis were further evaluated in this study.

## Methods

### Strains and media

*P. thermoglucosidasius* DSM 6285 was maintained and cultured routinely in modified Luria Bertani medium (mLB) [10]. Experimental cultures were grown in modified ammonium sulphate medium (mASM) [23].

### Experimental setup

Pre-cultures were grown in 100 mL mLB in 500 ml baffled shake flasks, inoculated with 40 $\mu {\text{L}}$ of glycerol stock and incubated aerobically at 55 °C for 14 h at 120 rpm in an Infors HT Thermotron (Infors AG, Bottmingen, Switzerland). Following incubation, appropriate volumes of pre-culture were used to inoculate experimental cultures to an initial OD_600_ of ~ 0.1. Experimental cultivations were conducted in 250 ml serum bottles sealed with a rubber stopper, containing 50 mL ASM with differing gas headspace compositions at atmospheric pressure.

To investigate the effects of variable industrial syngas mixtures on the *P. thermoglucosidasius* DSM 6285 WGS reaction, the strain was cultured in several gas headspace atmospheres (Table 1). Four sets of experimental cultures were cultivated in triplicate. The first set served as an aerobic control grown under an optimal gas atmosphere composed of 50% air and 50% CO. The second set served as an anaerobic control, with a gas headspace composed of 50% N_2_ and 50% CO. This was achieved by flushing bottle headspaces with N_2_ for 5 min at a flow rate of 0.2 L/minute, followed by injection of the bottles with CO to an overpressure of 1 bar, with subsequent equilibration to atmospheric pressure. The third culture set was cultured under an initial gas headspace comprising 50% N_2_ and 50% synthetic syngas mixture (Table 1; Air Liquide, Düsseldorf, Germany). This was achieved by flushing bottle headspaces with N_2_ for 5 min at a flow rate of 0.2 L/minute, then injecting the bottles with the synthetic gas mixture to an overpressure of 1 bar, followed by subsequent equilibration to atmospheric pressure. The fourth culture set was cultivated under a 100% atmosphere of the synthetic syngas mixture. Following inoculation, bottles were incubated at 55 °C for 134 h at 120 rpm.

**Table 1:** Composition of synthetic syngas stock and initial gas headspace mixtures

	Gas component (%)								
Atmosphere	CO_2_	CO	CH_4_	N_2_	H_2_	C_4_H_10_	C_2_H_6_	C_3_H_8_	O_2_
Synthetic syngas stock	~ 43.99	36.90 ± 0.07	6.22 ± 0.12	5.07 ± 0.10	3.64 ± 0.07	2.42 ± 0.05	1.03 ± 0.02	0.73 ± 0.01	0
Aerobic Control	0.42 ± 0.03	48.61 ± 0.11	0	39.73 ± 0.10	0	0	0	0	9.93 ± 0.73
Anaerobic (N_2_) Control	0.31 ± 0.05	45.51 ± 0.36	0	52.90 ± 0.44	0	0	0	0	0
Syngas 50% mixture*	17.08 ± 1.64	17.28 ± 0.77	3.91 ± 0.37	59.37 ± 2.02	1.05 ± 0.01	~ 1.21	~ 0.52	~ 0.37	0
Syngas 100% mixture*	41.65 ± 1.60	38.01 ± 0.92	6.30 ± 0.09	9.68 ± 0.50	3.11 ± 0.00	2.42 ± 0.05	1.03 ± 0.02	0.73 ± 0.01	0

*Theoretical composition inferred from dilution of stock gas

To investigate the influence of O_2_ addition on WGS reaction-driven hydrogenogenesis, two experiments were conducted. In all cases, the serum bottle gas headspace of each stoppered replicate was reconstituted to an ideal initial atmosphere of 50% air and 50% CO prior to inoculation [23]. This was achieved by flushing bottles with CO to an overpressure of 1 bar, then equilibrating the headspace to atmospheric pressure. O_2_ was injected into the gas headspace of bottles containing actively fermenting cultures at various stages of the fermentation. Four time points (post-inoculation) were selected for O_2_ addition, reflective of distinct periods of the WGS reaction fermentation profile [18]. These included the mid aerobic phase (14 h), the commencement of the anaerobic phase (24 h), the early-mid hydrogenogenic phase (38 h) and the late hydrogenogenic phase (48 h). The experiment was carried out in two batches, comprising three sets of cultures, one control and two experimental cultures, respectively. In each batch, cultures were cultivated in triplicate with one set serving as a control where no O_2_ was added at any timepoint. The two remaining culture sets in each batch were designated a time point where a defined volume of O_2_ was injected. O_2_ volumes injected were ~ 5% of the initial serum bottle gas headspace volume, calculated as ${v}_{{O}_{2}}={v}_{t}-{v}_{m}-{v}_{i}$, where ${v}_{{O}_{2}}$ = volume O_2_ added, ${v}_{T}$ = total serum bottle volume, ${v}_{m}$ = volume of the liquid culture medium and ${v}_{i}$ = inoculum volume. Following inoculation, bottles were incubated at 55 ˚C for 120 h at 120 rpm, with O_2_ being added to the appropriate replicate set at the designated time points.

Finally, the influence of different volume O_2_ additions on the WGS system at a single time point was investigated. A 38-h post-inoculation timepoint, representative of the peak hydrogenogenic period, was selected based on the fermentation profiles of the previous experiments. Five sets of experimental cultures were cultivated in triplicate under a gas atmosphere of 50% air and 50% CO, with one set serving as a control where no O_2_ was added. Each of the four remaining culture sets were designated an O_2_ volume to be injected at 38 h, either ~ 1, 3, 5 or 10% of the initial serum bottle gas headspace volume, calculated as described above. Following inoculation, bottles were incubated at 55 ˚C for 120 h at 120 rpm, with the appropriate O_2_ volumes being added to the respective replicate sets at 38 h.

### Sampling and analytics

Liquid culturing medium and gas headspace sampling was conducted at various time points for each experiment. For the experiment where different syngas mixtures were evaluated, samples were taken at 0, 14, 24, 38, 48, 62, 72, 86, 96, 110, 120 and 134 h post-inoculation. For the first oxygen supplementation experiment, where a fixed O_2_ volume was supplemented at various timepoints in the WGS reaction fermentation, sampling points were 0, 10, 14, 24, 34, 38, 48, 58, 72, 84, 96, 110 and 120 h post-inoculation. Sampling points for the final experiment, where variable O_2_ volumes were supplemented during the peak hydrogenogenic period were 0, 14, 24, 38, 41, 44, 48, 62, 72, 86, 96, 110, 120, and 134 h post-inoculation. In both O_2_ addition experiments, prior to the addition of O_2_ at applicable time points, both a liquid and gas sample were taken and immediately followed by a second gas sampling post-O_2_-addition. A volume of 3 mL gas headspace was drawn at each time point and analyzed using a 300 Micro GC gas analyzer (Inficon, Switzerland), with argon and helium as carrier gases connected with Molsieve 5 Å and PLOT Q columns for data acquisition. Gas composition was calculated based on the ideal gas law as previously described [10]. Headspace pressure was determined before sampling using a manometer (GDH 14 AN, Greisinger electronic, Regenstauf, Germany) and temperature was maintained at 60 ˚C. A volume of 1 mL of liquid culture medium was sampled at each sampling point for growth and used for subsequent HPCL analysis. Growth was estimated using absorbance (OD_600_) values using an Ultrospec 1100 pro spectrophotometer (Amersham Biosciences, Uppsala, Sweden) and pH was measured using a Profilab pH 597 (Xylem Analytics, Weilheim, Germany).

Following absorbance and pH measurement, samples were centrifuged at 16,000 × *g* for 10 min and supernatants transferred to a clean 1.5 mL microcentrifuge tube for storage at − 20 ˚C. Thawed supernatants (100 μL) were dispensed in 1.5 mL HPLC autosampler vials fitted with micro-inserts. Glucose and twelve central carbon metabolites (1-propanol, acetate, butyrate, ethanol, formate, glyoxylate, iso-butyrate, iso-valerate, lactate, propionate, succinate and valerate) were monitored over 0–2.0 g/L concentration ranges. Samples were analyzed using an Agilent 1100 series HPLC system (Agilent Technologies, Waldbronn, Germany) connected to a wavelength detector and refractive index detector with a 50 mm long pre-column (model Rezex ROA-Organic Acid H^+^ 8% Guard Column) and a 300 mm long separation column (model Rezex ROA-Organic Acid H^+^ 8%). Analysis was conducted using a 5 mM H_2_SO_4_ mobile phase, 50 ˚C column temperature, 0.6 mL/min flow rate for 40 min per sample and injection volume of 20 μL. Data acquisition and handling was conducted using Chemstation (Agilent Technologies) and datasets were summarized and figures plotted using R v. 4.3.2 [24, 25].

### Statistical analysis

Statistical analysis for the Gas chromatography and HPLC was performed in R v. 4.3.2 [24, 25]. To determine significant changes in gas headspace and metabolite profile compositions between the controls and experimental cultures, one way analysis of variance (ANOVA) was conducted between the respective constituents within each experiment, followed by post-hoc Tukey Honest Significant Difference (HSD) tests. To determine significant changes in H_2_ following O_2_ addition in both the temporal and volumetric experiments, H_2_ levels at each sampling point within culture sets were subjected to one way ANOVA, followed by post-hoc Tukey HSD tests.

## Results

### Anaerobic synthesis gas mimetic feedstocks promote earlier onset of hydrogenogenesis at the expense of biomass accumulation

**Fig. 1:**
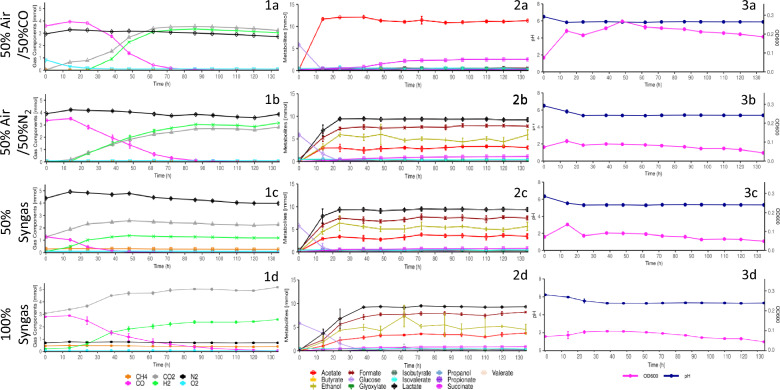
Gas composition (1), metabolite (2) and growth and pH (3) profiles for *P. thermoglucosidasius* DSM 6285 cultured under 50% CO/50% Air- (**a**) 50% CO/50% N_2_- (**b**) 50% synthetic syngas- (**c**) and 100% synthetic syngas- (**d**) -gas headspaces

Previous studies have characterised the fermentation profile of *P. thermoglucosidasius* DSM 6285 under various aerobic and anaerobic CO enriched headspaces, citing a 50% CO/50% air atmosphere as an ideal balance between suitable growth conditions and facilitating downstream WGS reaction conditions [18–20, 23]. Here we observed distinct fermentation profiles between the aerobic and anaerobic control, syngas (50%) and syngas (100%) atmospheres (Fig. 1).

The aerobic control growth profile corresponded with previously observed patterns. This included an initial sharp increase in absorbance during the aerobic phase, peaking at 0.327 ± 0.008 by 48 h (coincidental with peak WGS reaction activity), followed by gradual decreases towards the end of fermentation (Fig. 1). All anaerobic atmospheres exhibited significantly poorer growth throughout the fermentations (Additional file 1: Table S1). Slight, albeit much less pronounced increases were observed for the anaerobic control (50%) and syngas (50%) atmospheres during the first 14 h of the experiment. This growth was not, however, shared by the syngas (100%) atmosphere replicates, which increased negligibly, plateauing similarly to the N_2_ and synthetic syngas (50%) atmosphere replicates at 0.111 ± 0.007. Growth of the syngas (100%) atmosphere replicates was also atypical, exhibiting a granular flocculant-type of growth compared to the turbid/cloudy appearance observed in the other atmospheres (results not shown).

In the early phases of each fermentation, pH values decreased across all atmospheres to varying extents (Fig. 1). The aerobic control reached a low of 5.82 ± 0.02 by 14 h, accompanied and likely accounted for by concerted acetate production observed in this period. Anaerobic atmospheres demonstrated similar pH profiles to the aerobic control, however, reaching significantly lower pH values of 5.31 ± 0.04 by 38 h (Additional file 1: Table S1). These atmospheres exhibited strong concurrent production of lactate, formate and acetate which plateaued in conjunction with pH stabilisation and were likely responsible for the observed pH fluctuations. Following initial decreases, pH stabilized across all atmospheres, with the aerobic control stabilizing at a final value of 5.87 ± 0.02 and the anaerobic atmospheres at 5.37 ± 0.03.

H_2_ production was observed within 14 h in the anaerobic atmospheres (Fig. 1), while the aerobic control only showed H_2_ production by 24 h. Notably, H_2_ production in the aerobic control was observed with concurrent O_2_ levels of 0.95 ± 0.06%. Peak H_2_ productivity for the aerobic control was much higher than the anaerobic atmospheres with peak productivity observed between 38 and 48 h at 0.0137 ± 0.0048 mmol h^−1^. For the anaerobic control and syngas (100%) atmospheres, lower peak productivity was observed earlier between 24 and 38 h at 0.0063 ± 0.0005 mmol h^−1^ and 0.0047 ± 0.0016 mmol h^−1^ respectively, while the syngas (50%) atmosphere exhibited the earliest peak productivity between 14 and 24 h at 0.0055 ± 0.0005 mmol h^−1^. Notably for the anaerobic atmospheres, H_2_ production appeared to coincide with mixed acid fermentation, suggesting concurrent operation of these processes (Fig. 1).

H_2_ mol yields per CO mol consumed (H_2_/CO) across the fermentations varied between the points where CO was completely consumed and the endpoints of the fermentations at 134 h. Each atmosphere, except for the syngas (100%) atmosphere showed complete CO consumption within 134 h. Unsurprisingly, due to its comparatively low initial CO levels, the syngas (50%) atmosphere demonstrated the earliest CO depletion (84 h), followed by the aerobic control (110 h), then the anaerobic control (120 h). By 134 h, 1.06 ± 0.63% CO remained in the syngas (100%) atmosphere gas headspace. At the points where CO was completely consumed, the aerobic and anaerobic controls and the syngas (50%) atmosphere exhibited significantly different H_2_/CO yields from one another (Additional file 1: Table S2), of 89.48 ± 0.83%, 85.40 ± 0.14% and 93.62 ± 0.31% respectively. However, by the end point of the fermentations, yields fluctuated further, with both the aerobic control and syngas (50%) atmospheres having exhibited decreases of 4.92 ± 0.47% and 6.73 ± 0.85% respectively. Conversely, the anaerobic control H_2_/CO yield increased by 8.48 ± 0.43%. By 134 h, despite not having depleted CO, the H_2_/CO yield of the syngas (100%) atmosphere stood at 83.83 ± 1.42%, similar to that of the aerobic control but significantly lower than those of the anaerobic control and syngas (50%) atmospheres (Additional file 1: Table S2).

Analysis of the metabolite profile with HPLC showed that lactate, formate and ethanol were, in descending order, the most prevalent metabolites produced under anaerobic atmospheres, while only negligible amounts of formate and ethanol were observed for the aerobic atmosphere (Fig. 1). Lactate, formate and ethanol levels across the anaerobic atmospheres stabilized at 9.3442 ± 0.2808 mmol, 7.6075 ± 0.4817 mmol and 5.2032 ± 1.0371 mmol after 38 h levels despite the syngas (100%) atmosphere having exhibited significantly lower productivity of each acid between 0–14 h (Additional file 1: Table S1).

Acetate was the central metabolite detected through HPLC in the aerobic atmosphere, rising rapidly and peaking at 12.1255 ± 0.2620 mmol by 38 h (Fig. 1). Slight decreases in acetate levels were observed between 38 and 62 h to 11.0224 ± 0.1368 mmol but remained relatively stable thereafter. Acetate accumulation in the anaerobic atmospheres was significantly lower than the aerobic atmosphere (Additional file 1: Table S1), accumulating to only a third of that of the aerobic control. The aerobic control also exhibited significant increases in succinate production between 38 and 72 h (Additional file 1: Table S1), reaching a final level of 2.5111 ± 0.0606 mmol, while the anaerobic atmospheres showed negligible succinate accumulation. Glucose was completely consumed within 14 h post-inoculation in the aerobic control. Low levels of glucose, below the 0.1 mg L^−1^ HPLC method detection limit, were observed throughout all three anaerobic fermentations. However, most of the initially available glucose (> 99%) was consumed by 24 h in the N_2_ control and syngas (50%) atmospheres and by 38 h in the syngas (100%) atmosphere.

### Pre-hydrogenogenic phase O_2_ perturbations extend the lag phase prior to H_2_ production

**Fig. 2:**
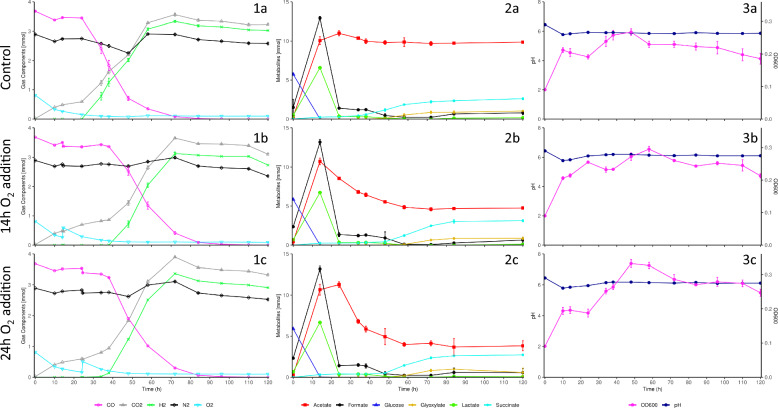
Gas composition (1) metabolite (2) and growth and pH (3) profiles for *P. thermoglucosidasius* DSM 6285 cultured under a 50% CO/50% Air gas headspace with no O_2_ addition (**a**), 14 h O_2_ addition (**b**) and 24 h O_2_ addition (**c**)

**Fig. 3:**
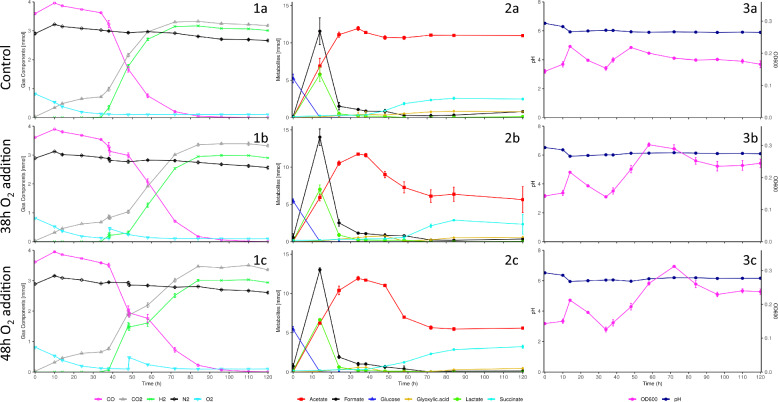
Gas composition (1), metabolite (2) and growth and pH (3) profiles for *P. thermoglucosidasius* DSM 6285 cultured under a 50% CO/50% Air gas headspace with no O_2_ addition (**a**), 38 h O_2_ addition (**b**) and 48 h O_2_ addition (**c**)

Oxygen addition at the various timepoints resulted in distinct fermentation profiles. Oxygen additions at 14 h and 24 h (Fig. 2) exhibited markedly different influences on the various fermentation profiles compared to the control and 38 h and 48 h O_2_ additions (Fig. 3). For all O_2_ additions, significantly higher peak and final absorbance (OD_600_) values were observed compared to the controls following O_2_ addition (Additional file 1: Tables S3 and S4). In the cases of the 38 h and 48 h O_2_ additions, which coincided with the hydrogenogenic phase, severe reductions in H_2_ production were observed, implying the growth observed was primarily the result of O_2_ addition and not supplemented through the WGS reaction. While the control OD_600_ values declined following peak levels at 48 h, the 38 h and 48 h O_2_ additions exhibited further significantly elevated peak OD_600_ values of 0.332 ± 0.011 and 0.302 ± 0.006 at 48 h and 58 h respectively following oxygen addition (Additional file 1: Tables S3 and S4).

Steep declines in pH to lows of 5.78 ± 0.01 (Fig. 2) and 5.93 ± 0.02 (Fig. 3) within 14 h were observed across the fermentation batches in the aerobic phase, reflective of the observed acetate, formate and lactate accumulation. In the controls, these declines were followed by slight increases alongside near complete and complete consumption of formate and lactate, stabilizing at final pH values of 5.88 ± 0.02. Similar profiles were observed until either the addition of O_2_ in the 14 h and 24 h O_2_ addition replicates or 34–38 h in the 38 h and 48 h O_2_ additions replicates. O_2_ addition resulted in the significant increase in pH compared to the controls (Additional file 1: Tables S3 and S4), until the final sampling point, likely the result of decreases in observed acetate levels.

H_2_ gas was first detected across all samples within 38 h. The highest O_2_ level detected with concurrent H_2_ production was 2.49 ± 0.06% O_2_. O_2_ additions elevated O_2_ levels an average of 4.51 ± 0.35%. Peak H_2_ productivities were experienced between 34 and 38 h at 0.0297 ± 0.0004 mmol h^−1^ (Batch 1 control), 0.0205 ± 0.0045 mmol h^−1^ (Batch 2 control) and 0.0130 ± 0.0052 mmol h^−1^ (38 h O_2_ addition). The 48 h O_2_ addition replicates demonstrated peak H_2_ productivity between 38 and 48 h at 0.0143 ± 0.0006 mmol h^−1^, whilst both the 14 h and 24 h O_2_ additions showed more delayed peak productivities of 0.0133 ± 0.0004 mmol h^−1^ and 0.0123 ± 0.0002 mmol h^−1^, respectively, between 48 and 58 h. H_2_ productivity decreased gradually as CO was depleted, eventually resulting in the net consumption of H_2_, with a maximal H_2_ consumption rate of 0.0030 ± 0.0002 mmol h^−1^ across all replicate sets.

Significant increases in H_2_ levels were observed in both controls between 24 and 72 h (Additional file 1: Table S5). In each instance where O_2_ was added, significant disruptions to H_2_ production were observed (Additional file 1: Tables S3 and S4). H_2_ production was delayed following O_2_ addition at 14 h and 24 h and resulted in significantly lower final H_2_ levels (2.7351 ± 0.0058 mmol and 2.9094 ± 0.0127 mmol respectively) compared to the control (3.0260 ± 0.0112 mmol). H_2_ productivity was significantly lower between both O_2_ additions and the control between 24 and 38 h. Notably, the 24 h O_2_ addition replicates showed earlier and more intensive H_2_ production than the 14 h O_2_ addition replicates, with comparably higher H_2_ levels and H_2_ productivities during the majority of the fermentation (Fig. 2; Additional file 1: Tables S3 and S4). Following O_2_ addition for the 38 h and 48 h O_2_ addition replicate sets, both H_2_ levels and H_2_ productivities were significantly lower than the control for the subsequent 10 h period (Additional file 1: Tables S3 and S4). However, weak H_2_ production was still observed across this period, with H_2_ productivities of 0.0011 ± 0.0004 mmol h^−1^. Following this period, however, significant increases in H_2_ levels were observed for the 38 h and 48 h O_2_ addition replicates between 48–84 h and 58–84 h respectively (Additional file 1: Table S5). However, H_2_ levels for both O_2_ additions remained significantly lower than the control following O_2_ addition.

Both controls and pre-hydrogenogenic phase 14 h and 24 h O_2_ additions showed complete utilisation of CO, with trace amounts of CO remaining in the 38 h and 48 h O_2_ addition replicates (Fig. 2). H_2_/CO yields at the points of complete CO depletion were 83.26 ± 1.19% (Controls), 74.35 ± 0.07% (14 h O_2_ addition) and 79.10 ± 0.26% (24 h O_2_ addition), respectively. These were all significantly different from one another (Additional file 1: Table S2). Yields of 80.35 ± 0.13% and 81.33 ± 0.30% were observed for the 38 h and 48 h O_2_ addition replicates by 120 h. Both yields were significantly lower than the respective control but not significantly different from one another (Additional file 1: Table S2).

HPLC analysis detected fluctuations in acetate and succinate profiles following oxygen addition. Acetate levels across all samples increased dramatically from the onset of the fermentations and peaked either prior to O_2_ addition or within 34 h post-inoculation (Fig. 2). In the controls, after reaching peak acetate levels of 11.4257 ± 0.5833 mmol, a low-level net consumption of acetate was observed, gradually reducing acetate levels to a final concentration of 10.4132 ± 0.6191 mmol. In each case where O_2_ was added, significantly lower acetate levels were observed throughout the remainder of the fermentations compared to the respective controls (Additional file 1: Tables S3 and S4). Correspondingly, significantly higher acetate consumption levels were observed between all O_2_ addition replicates and the controls immediately following O_2_ addition. This was observed between 14–34 h (14 h O_2_ addition), 24–38 h (24 h O_2_ addition), 38–82 h (38 h O_2_ addition) and 38–72 h (48 h O_2_ addition), respectively post-O_2_ addition.

Although succinate levels were observed to increase gradually from the start of all fermentations, prominent increases were only observed after 24 h in the controls, peaking at 2.544 ± 0.1405 mmol (Fig. 2). O_2_ addition at 14 h and 24 h delayed succinate accumulation, presenting significantly lower succinate levels than the control between 34–58 h and 38–58 h respectively (Additional file 1: Table S3). Despite this, the 14 h O_2_ addition replicates accumulated significantly higher final succinate levels compared to both the control and 24 h O_2_ addition replicates, peaking at 3.1261 ± 0.1344 mmol. Following O_2_ addition at 38 h and 48 h, significantly lower levels of succinate were observed compared to the control between 48–58 h (Additional file 1: Table S4). Despite this delay in succinate production, there were no significant differences between the final accumulated succinate concentrations of the control, 38 h and 48 h O_2_ additions, which averaged at 2.7774 ± 0.9710 mmol.

Glucose levels across all samples were almost completely consumed (> 97%) 14 h post-inoculation and were completely consumed within 34 h. Large formate 12.9833 ± 1.0624 mmol and lactate 6.5760 ± 0.5515 mmol peaks were detected at 14 h post-inoculation across each replicate set. Both compounds were almost completely consumed thereafter with only minor increases in formate below the detection limit (< 0.1 g/L) of the HPLC method.

### Minor O2 perturbation during peak-hydrogenogenesis minimally disrupts H2 production in *P. thermoglucosidasius* DSM 6285

**Fig. 4:**
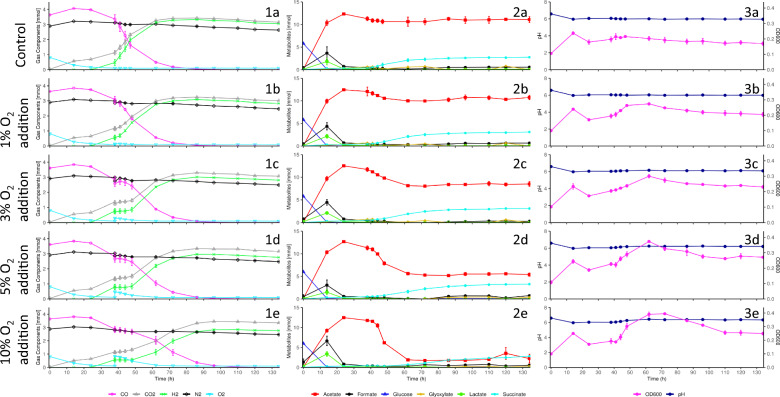
Gas composition (1) metabolite (2) and growth and pH (3) profiles for *P. thermoglucosidasius* DSM 6285 cultured under a 50% CO/50% Air gas headspace with no O_2_ addition (**a**), 1%- (**b**), 3%- (**c**), 5%- (**d**) and 10%- (**e**) -O_2_ addition at 38 h

Prior to oxygen addition, the 38 h varied O_2_ volume addition fermentations demonstrated the same trends in growth, pH, gas composition and HPLC metabolite profiles as observed previously (Figs. 1, 2). Following varied oxygen additions, however, disruptions in these profiles were scaled accordingly with the volumes of oxygen supplemented (Fig. 4). O_2_ levels post-O_2_ addition were 0.90 ± 0.22% (1% O_2_ addition), 2.83 ± 0.07% (3% O_2_ addition), 4.70 ± 0.14% (5% O_2_ addition) and 9.08 ± 0.14% (10% O_2_ addition), respectively.

Following O_2_ addition OD_600_ values increased in accordance with volumes of O_2_ added, with larger O_2_ volume additions resulting in greater peak OD_600_ values achieved (Fig. 4). The 1% O_2_ addition prompted the weakest response, but exhibited significantly higher values compared to the control from 48 h onwards (Additional file 1: Table S5). The 3% O_2_ addition showed a slightly more delayed increase in OD_600_ values, with significantly elevated OD_600_ values compared to the control from 62 h onwards. The 5% and 10% O_2_ additions both exhibited a slight decrease in OD_600_ values following O_2_ addition between 38 and 41 h, however, showed a steep increase thereafter. From 44 h onwards, the 5% O_2_ addition maintained significantly higher OD_600_ values compared to the control. The 5% O_2_ addition also maintained significantly higher OD_600_ values than those of the 1% and 3% O_2_ additions over the majority of the fermentation and was similar to the 10% O_2_ addition. Although the 10% O_2_ addition exhibited the greatest increase in OD_600_ values, peaking at 0.395 ± 0.006 at 72 h, OD_600_ values appeared to decrease sooner than those of the 5% O_2_ addition.

Larger O_2_ volume additions correspondingly resulted in larger sustained increases in pH, likely the result of the observed reduction in acetate levels following O_2_ addition (Fig. 4). Apart from the 1% O_2_ addition, which displayed no significant difference from the control, all other O_2_ additions prompted significant increases in pH values 6 h post-O_2_ addition (Additional file 1: Table S5). pH values stabilized in each case after ~ 62 h, alongside plateaus in acetate consumption and OD_600_ peaks, with the 10% O_2_ addition almost returning to starting pH values, reaching as high as 6.35 ± 0 (Fig. 4).

H_2_ was first detected at 38 h across all replicate sets at elevated levels (Fig. 4). The control exhibited a H_2_ productivity of 0.0460 ± 0.0013 mmol h^−1^ between 38 and 41 h. Comparatively, H_2_ productivities of the O_2_ addition replicates were all significantly lower than the control in this period (1%: 0.0137 ± 0.0062 mmol h^−1^; 3%: 0.0002 ± 0.0003 mmol h^−1^; 5%: 0.0005 ± 0.0012 mmol h^−1^; 10%: 0.0009 ± 0.0003 mmol h^−1^; Additional file 1: Table S5). In the case of the 1% O_2_ addition, no significant difference in H_2_ productivity with the control was detected after 41 h (Additional file 1: Table S6), with H_2_ productivity between 38–48 h (0.0101 ± 0.0013 mmol h^−1^), the peak H_2_ productivity period, only slightly lower than that of the control (0.0150 ± 0.0006 mmol h^−1^). However, unlike the control, no significant increase in H_2_ levels were observed between 38 and 41 h (Additional file 1: Table S5). Significant increases in H_2_ levels were only detected in the 1% O_2_ addition replicates between 41 and 62 h (Additional file 1: Table S5), when O_2_ levels were below 0.79 ± 0.13%. Despite this, no significant difference in H_2_ concentration was detected between the 1% O_2_ addition and the control over this period (Additional file 1: Table S6). However, following complete CO consumption, the 1% O_2_ addition replicates exhibited significantly lower final H_2_ levels (2.8999 ± 0.0788 mmol) than the control (3.1277 ± 0.0874 mmol; Additional file 1: Table S6).

Following O_2_ addition, significantly lower levels of H_2_ were detected between the 3%, 5% and 10% O_2_ addition replicates and the control until the endpoints of the fermentations (Additional file 1: Table S6). Lower final H_2_ levels were observed accordingly with higher volumes of O_2_ added (Control: 3.0525 ± 0.0110 mmol, 1% O_2_ addition: 2.8290 ± 0.0097 mmol, 3% O_2_ addition: 2.8038 ± 0.0084 mmol, 5% O_2_ addition: 2.7613 ± 0.0085 mmol, 10% O_2_ addition: 2.7657 ± 0.0096 mmol). However, no significant differences in final H_2_ levels were observed between the 1% and 3%- and between the 5% and 10% -O_2_ addition replicates (Additional file 1: Table S6). Following the reduction in H_2_ productivity experienced by the 3%, 5% and 10% O_2_ addition replicates between 38 and 41 h, H_2_ consumption was observed for each of these O_2_ additions between 41 and 44 h at rates of 0.0002 ± 0.0005 mmol h^−1^, 0.0019 ± 0.0002 mmol h^−1^ and 0.0018 ± 0.0005 mmol h^−1^ respectively (Fig. 4). H_2_ productivity for these replicates remained significantly lower than the control between 38 and 48 h for 3% and 5% O_2_ addition replicates and 38–62 h for the 10% O_2_ addition replicates (Additional file 1: Table S6). However, H_2_ productivity increased following 44 h with the 10% O_2_ addition replicates demonstrating significantly higher (P < 0.01) H_2_ productivity than the control between 62 and 96 h (Additional file 1: Table S6). Consequently, significant increases in H_2_ levels were only detected between 48 and 62 h for the 3% and 5% O_2_ addition replicates and between 62 and 72 h for the 10% O_2_ addition replicates (Additional file 1: Table S5). Following O_2_ addition, O_2_ levels at the first point where a significant increase in H_2_ was observed (Additional file 1: Table S6), were 0.03 ± 0.06% (3% O_2_ addition), 0.17 ± 0.09% (5% O_2_ addition) and 0.97 ± 0.1% (10% O_2_ addition).

CO was completely consumed across each condition by 134 h (Fig. 4). The control exhibited CO depletion first (110 h) alongside the 1% O_2_ addition replicates, then the 3% O_2_ addition replicates (120 h) and the 5% and 10% O_2_ addition replicates (134 h). The control exhibited the highest H_2_/CO yield both at the first point of CO depletion (88.56 ± 0.62%) and by the end of the fermentation (83.86 ± 0.62%). In both instances yields were significantly higher than all O_2_ addition yields (Additional file 1: Table S2), despite exhibiting a decrease in yield following CO depletion. Although producing significantly different yields at the first points of CO depletion (82.26 ± 0.25% and 85.26 ± 0.30% respectively), the 1% and 3% O_2_ additions showed no significant difference in H_2_/CO yield by the endpoint of the fermentation (Additional file 1: Table S2), with final yields of 77.82 ± 0.00%. In both cases, however, reductions in H_2_/CO yields of 4.70 ± 0.41% and 8.30 ± 0.94%, respectively, were observed between the two points. The 5% followed by 10% O_2_ additions exhibited the lowest H_2_/CO yields at 134 h, with yields of 76.51 ± 0.20% and 75.89 ± 0.24%, respectively.

Acetate levels across all samples increased dramatically from the onset of the fermentations and peaked prior to O_2_ addition (Fig. 4). Peak acetate levels of 12.5025 ± 0.1713 mmol were observed by 24 h, followed by net reduction, with the control reaching a final acetate concentration of 11.1013 ± 0.6522 mmol. The 1% O_2_ addition showed almost no differences in acetate levels compared to the control (Additional file 1: Table S6). In the cases of the 3%, 5% and 10% O_2_ additions, significantly lower acetate levels were observed after 48 h, 44 h and 48 h, respectively, with final acetate levels of 8.5019 ± 0.5227 mmol (3% O_2_ addition), 5.3856 ± 0.3675 mmol (5% O_2_ addition) and 2.2098 ± 1.0733 mmol (10% O_2_ addition). Greater degrees of acetate consumption were observed in accordance with greater volumes of O_2_ added, with the most noteworthy decreases in acetate observed by the 10% O_2_ addition, followed by the 5%, 3% and finally 1% O_2_ additions, with the latter showing no significant difference from the control (Additional file 1: Table S6).

Succinate levels increased gradually from the start of all fermentations (Fig. 4), although prominent increases in succinate were staggered. Whilst there was no significant difference in succinate productivity immediately following O_2_ addition between the 1%, 3% and 5% O_2_ additions and the control, the 10% O_2_ addition exhibited net succinate consumption following O_2_ addition, between 38 and 48 h and significantly lower succinate productivity until 62 h post-inoculation (Additional file 1: Table S6). All O_2_ additions exhibited significantly higher succinate productivity compared to the control between 62 and 

–84 h. Compared to the control which exhibited final succinate levels of 2.7534 ± 0.1376 mmol, the 5% O_2_ addition replicates accrued significantly higher levels of 3.2737 ± 0.2034 mmol by the end of the fermentation. Glucose levels across all samples were almost completely consumed (> 97%) within 14 h and were completely consumed within 34 h. Formate and lactate peaks were detected at 14 h post-inoculation across each replicate set, however, both compounds were almost completely consumed thereafter with only minor increases in formate below the detection limit (< 0.1 g/L) of the HPLC method.

## Discussion

Syngas fermentation provides the opportunity for microbial conversion to various value-added products from industrial waste gases [26]. Here we explored the effects of synthetic syngas mixtures on the *P. thermoglucosidasius* WGS reaction. Our analyses showed that *P. thermoglucosidasius* grown in anaerobic syngas atmospheres, with highly varied CO and CO_2_ compositions and impurities including methane, ethane, propane and butane, was able to produce H_2_, being limited only by CO availability. However, cultivation in these anaerobic atmospheres severely hampered biomass accumulation.

Poor biomass accumulation was likely due to the distinct bioenergetics associated with aerobic and anaerobic respiration as well as fermentation. Anaerobic atmospheres exhibited distinct mixed acids fermentation profiles favouring lactate, formate, ethanol and acetate production, which could account for the more marked reductions in pH observed across these conditions [27–30]. The aerobic control exhibited pronounced acetate production prior to commencement of H_2_ production, alongside concerted succinate production which coincided with O_2_ depletion and onset of the WGS reaction. As well as serving as a means for balancing cell redox levels and potentially as an alternative carbon pool, it has been suggested that elevated acetate levels increase the CO uptake capacity of CO consumers [23, 31]. Notably, two-fold greater H_2_ production rates were observed under an aerobic atmosphere, where acetate levels were also several fold higher than anaerobic/microaerobic atmospheres.

In the syngas atmospheres, varying CO levels influenced growth and metabolism to a large extent, impacting downstream H_2_ production. H_2_ production in these atmospheres showed considerably shorter lag phases between inoculation and onset of the WGS reaction, similar to the anaerobic control and previous anaerobic cultivations that have exhibited reductions to within 4 h post-inoculation [19]. The 50% syngas, atmosphere, with the lowest CO level (~ 17%), exhibited pronounced earlier onset of fermentative metabolism and H_2_ production compared to the anaerobic control (CO level ~ 46%) and the syngas (100%) atmosphere (CO level ~ 38%). From previous aerobic studies the opposite was anticipated as atmospheres with enriched CO levels facilitated more prolific growth responses and correspondingly greater hydrogenogenic activity [20, 23]. We observed distinct conversion efficiencies between the first observed points of CO depletion and the fermentation end-points, with general yield decreases towards the end. Given the absence of known CO producing pathways, this was most likely the result of H_2_ consumption by one or both of two putative NiFe-uptake hydrogenases encoded on the genome of *P. thermoglucosidasius* DSM 6285 [17]. The consumption of H_2_ by group 1d uptake hydrogenases is coupled with aerobic respiration or respiratory reduction of anaerobic oxidants like fumarate, NO^3−^, SO_4_^2−^ and CO_2_ [12]. Group 2a uptake hydrogenases are involved in aerobic respiration and recycling H_2_ produced through nitrogenase activity or fermentative pathways in aerobic soil and Cyanobacteria [12]. It is plausible that these enzymes couple H_2_ oxidation with an anaerobic electron transport chain, however, this requires further validation.

Despite showing accelerated commencement of H_2_ productivity, anaerobic cultivation greatly reduced biomass accumulation, inadvertently reducing H_2_ production potential. It can be reasoned that sustained active biomass accumulation and consequently O_2_ availability are important considerations for developing this system towards more continuous operation. It would also be beneficial to balance maximally anaerobic conditions to minimally disrupt or delay WGS reaction onset, but maintain O_2_ supply to sustain catalytically active biomass. As such we further explored the impacts, both temporally and volumetrically, of O_2_ perturbation of the *P. thermoglucosidasius* DSM 6285 WGS reaction system at different key phases.

O_2_ addition across the different phases of the *P. thermoglucosidasius* DSM 6285 fermentation negatively impacted H_2_ production at all stages. O_2_ addition prior to commencement of H_2_ production delayed H_2_ production to a greater extent than O_2_ addition during the anaerobic phase, likely by extending more energetically efficient aerobic respiration [32]. O_2_ addition in the anaerobic phase likely prompted resumption of aerobic respiration, although, with a more limited pool of reducing agents in higher oxidation states, required more rapid commitment to the WGS reaction for energy conservation, as observed for the WGS system in other taxa [13, 33–36].

Later O_2_ additions in the hydrogenogenic phase showed stark reductions in H_2_ production alongside strong and extended gains in biomass accumulation, indicative of redirection of *P. thermoglucosidasius* towards aerobic metabolism, which offers greater potential energy yields. Expectedly, increasing volume O_2_ additions saw more pronounced disruptions to H_2_ production, however H_2_ production resumed, albeit at lower rates, following O_2_ depletion. As CO availability was not a limiting factor, this was likely the combined result of the extended effects of O_2_ induced inhibition of the WGS components and the shift towards maximizing use of the oxidative respiratory chain [15, 35, 37, 38]. The Ni-CODH and group 4a NiFe-hydrogenase responsible for conducting the *P. thermoglucosidasius* DSM 6285 WGS are both classed as highly O_2_ sensitive [12, 15, 35], although the tolerance of the *P. thermoglucosidasius* WGS system to O_2_ disruptions have not previously been investigated. Here we observed the initial commencement of WGS activity in the presence of 2.49 ± 0.06% O_2_ in the gas headspace and resumption of H_2_ production following O_2_ disruption once O_2_ levels dropped below 0.97 ± 0.1%.

Although 1% O_2_ addition initially reduced H_2_ productivity between 38 and 41 h, there were only minor observable differences in productivity of the 1% O_2_ addition condition (0.0101 ± 0.0013 mmol h^−1^) compared to the control (0.0150 ± 0.0006 mmol h^−1^) over the peak period of H_2_ productivity (38–48 h). Productivity remained positive throughout, despite O_2_ addition, demonstrating a final H_2_ yield with no significant difference to the control. Conversely, reductions in H_2_ levels were observed in the 3–10% O_2_ additions, most likely due to the activity of one or both of the two putative O_2_-tolerant NiFe-uptake hydrogenases, which potentially couple H_2_ oxidation with aerobic respiration.

The lag in growth experienced post 5% and 10% O_2_ addition coincided with relatively low increases in CO_2_, despite rapid consumption of O_2_, likely the result of the sudden pronounced oxidative stress. This phenomenon has been observed previously in *Klebsiella aerogenes* and *Escherichia coli*, where similar stimuli resulted in apparent uncoupling between growth and energy conservation, resulting in a switch towards energetically wasteful oxidation pathways [39, 40]. In conjunction with the O_2_ susceptibility of the WGS machinery, this may have effected lower H_2_ production and yields following both temporally and volumetrically diverse O_2_ perturbations.

Large increases in acetate, formate and lactate were detected early in all fermentations (Figs. 1, 2, 3, 4), resulting in pH decreases. These compounds likely served as overflow metabolites, acting as electron sinks to reduce the backlog of NADH generated from CO disrupted aerobic metabolism, at the expense of energetic efficiency [23, 28, 41]. Subsequently, shortfalls in ATP generation were likely compensated by acetate kinase-mediated acetate production. Accumulation of these acids have also been attributed to the development of microoxic conditions developed in the media during O_2_ consumption [42, 43]. Subsequent consumption of lactate was likely through the putative LutA-C protein complex encoded by *P. thermoglucosidasius*, proteins associated with aerobic lactate utilisation in *Bacillus subtilis* [23, 44]. Presently the fate of the carbon and electrons derived from the lactate remains unclear. Notably, formate was almost completely consumed by the onset of the WGS. Formate consumption is associated with concomitant CO_2_ and H_2_ production, however, no concomitant H_2_ production was observed in this period, suggesting diversion of electrons towards another oxidant or electron transport chain. It remains unclear as to how formate was metabolised in these circumstances and represents an area for further investigation.

Strong acetate production in the aerobic phase resembled previous *P. thermoglucosidasius* fermentations and likely compensated for detraction of potential ATP production by lactate and formate production [23, 28]. Acetate production was likely also bolstered through mixed acids fermentation to the detriment of ethanol-directed fermentation, which aligns with the insubstantial levels of ethanol detected here and in a previous study [28]. Following O_2_ addition at all timepoints strong acetate consumption accompanied by slightly delayed growth responses, increasingly so for larger volume O_2_ additions. Notably, greater degrees of CO_2_ than H_2_ were evolved between O_2_ addition and concerted resumption of WGS activity. Furthermore, following O_2_ addition, increases in succinate were observed towards the tail-end of acetate consumption, where O_2_ levels were approaching depletion. Together these factors suggest that a portion of the acetyl-CoA derived from the acetate consumed may have integrated into the TCA cycle and been incorporated into biomass via the glyoxylate shunt, as suggested previously [23, 45] however confirmation of this requires further validation. Following O_2_ depletion, acetate consumption ceased, indicating a necessity for O_2_ for concerted acetate consumption in *P. thermoglucosidasius*. Further investigation into the metabolism of *P. thermoglucosidasius* under these conditions may further yield a greater understanding of the dynamics and interplay between the WGS reaction and alternative metabolic pathways.

## Conclusions

Cultivation of *P. thermoglucosidasius* DSM 6285 under anaerobic synthetic syngas atmospheres induced faster onset of H_2_ production than previously identified ideal aerobic gas mixtures, however, this was at the expense of biomass accumulation. This in turn severely hampered H_2_ turnover. O_2_ supplementation at all stages of the fermentation hindered H_2_ production to greater extents accordingly with the volume of O_2_ supplied, however, also supported reinvigorated biomass accumulation. Furthermore, upon depletion of the supplemented O_2_, hydrogenogenic activity was rapidly resumed following a negligible lag period. Supplementation with 1% O_2_ negligibly impacted H_2_ production, however, still gave some support to growth. Notably, with elevated (10%) O_2_ supplementation, H_2_ consumption was observed briefly. This was potentially due to the two putative uptake hydrogenases encoded by *P. thermoglucosidasius* DSM 6285, which may present appealing targets for knockout mutagenesis. H_2_ conversion efficiency was also reduced accordingly with the level of O_2_ administered. The tolerance of the system to O_2_ perturbations may be linked with the metabolism of accumulated acetate, where O_2_ addition induces strong acetate consumption to support cell growth at all stages of the fermentation, including during the aerobic/microaerobic phase. It may be possible to incorporate this capacity for O_2_ induced acetate consumption and the relative flexibility of the *P. thermoglucosidasius* DSM 6285 WGS reaction to O_2_ perturbation to facilitate cyclical/fed batch hydrogenogenic fermentations on syngas at a larger scale. Additionally, focusing the strain towards concerted acetate production through inactivation of formate or lactate production pathways may facilitate concentrated acetate production to further supplement downstream acetate dependent growth.

### Supplementary Information

**Additional file 1: Table S1.** Significant differences observed between different initial gas headspace compositions. **Table S2.** Significant differences observed between CO/H_2_ yields. **Table S3.** Significant differences observed between pre-hydrogenogenic phase O_2_ addition conditions. **Table S4.** Significant differences observed between hydrogenogenic phase O_2_ addition conditions. **Table S5.** Significant differences in H_2_ levels observed between sampling points. **Table S6.** Significant differences observed between variable O_2_ additions during peak hydrogenogenic period.

## Data Availability

The dataset supporting the conclusions of this article is included within the article and its additional file.
